# On the Way to Understanding the Interplay between the RNA Structure and Functions in Cells: A Genome-Wide Perspective

**DOI:** 10.3390/ijms21186770

**Published:** 2020-09-15

**Authors:** Angelika Andrzejewska, Małgorzata Zawadzka, Katarzyna Pachulska-Wieczorek

**Affiliations:** Institute of Bioorganic Chemistry, Polish Academy of Sciences, Department of Structure and Function of Retrotransposons, Noskowskiego 12/14, 61-704 Poznan, Poland; aandrzejewska@ibch.poznan.pl (A.A.); mzawadzka@ibch.poznan.pl (M.Z.)

**Keywords:** transcriptome-wide studies, RNA structure in cells, structure-function relationship, translation, RNA stability and degradation, RNA binding proteome, RNA modifications

## Abstract

RNAs adopt specific structures in order to perform their biological activities. The structure of RNA is an important layer of gene expression regulation, and can impact a plethora of cellular processes, starting with transcription, RNA processing, and translation, and ending with RNA turnover. The development of high-throughput technologies has enabled a deeper insight into the sophisticated interplay between the structure of the cellular transcriptome and the living cells environment. In this review, we present the current view on the RNA structure in vivo resulting from the most recent transcriptome-wide studies in different organisms, including mammalians, yeast, plants, and bacteria. We focus on the relationship between the mRNA structure and translation, mRNA stability and degradation, protein binding, and RNA posttranscriptional modifications.

## 1. Introduction

RNA molecules fulfill a number of important functions. They take part in not only gene expression, as the intermediate between the DNA and protein, but also in many other cellular processes. It has emerged that the RNA structure, which single stranded RNA can fold into [1], adds another layer of information needed for the regulation of mechanisms such as the transcription, post-transcriptional processing, translation and protein folding, cellular localization, stability, and decay of RNA [2,3,4,5,6]. Although in vitro studies have enabled determination of the secondary structure of many RNAs, and the majority of the RNA secondary structure information is encoded within RNA sequence [7], plenty of results have shown that the RNA structures determined in vitro are not fully consistent with those in the cellular environment [4,8,9,10,11]. Thus, studying RNA architecture and its interactions with other RNA or protein partners in living cells has become fundamental to understanding the biochemical pathways of RNA acting.

**Figure 1:**
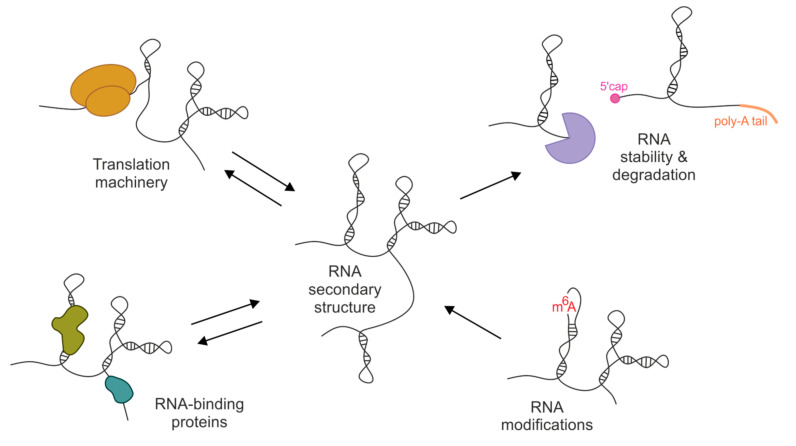
Schematic view on the relationship between the RNA secondary structure and cellular environment elements.

In the last decade, the development of new, sophisticated methods for the measurement of RNA structures in living cells has revolutionized the field of structural and functional RNA studies. Coupling RNA chemical probing in vivo with next generation sequencing and advanced bioinformatics tools has allowed for shifting the perspective from low-throughput studies to thousands of heterogeneous RNAs, and even whole transcriptomes, in complex cellular environments (reviewed in [12,13,14,15]). First genome-wide studies have been published for plant [8] and yeast [9] transcriptomes, and have suggested that RNAs are highly unfolded in vivo. Nevertheless, with the increasing number of in vivo studies, a view of the cell RNA structure as “wet spaghetti” has been displaced [16]. A growing amount of evidence has indicated that the RNA structure can be affected by various cellular factors, but RNA still contains well-defined structural motifs that often display important regulatory functions (Figure 1). The interplay between the cellular environment and RNA structure has become an important question that needs to be resolved.

Here, we present the current view on the RNA secondary structure in vivo resulting from the most recent transcriptome-wide studies. We summarize and discuss new findings about the impact of cellular factors on the folding of RNA and the relationship between RNA structure and its functioning in the cell. We focus on the correlation between RNA structure and translation, protein binding, RNA stability, and degradation and the role of RNA modifications in the folding of RNA molecules in the cell. However, this review also shows that despite the growing number of transcriptome-wide RNA structural studies in vivo, there are still large gaps in the understanding of RNA folding in living cells.

## 2. Approaches for RNA Structure Determination

For a long time, our knowledge on RNA secondary structure was based mainly on in silico methods that calculate the most thermodynamically favorable states of RNA or predict the consensus structures conserved in multiple homologous RNA sequences [17,18,19,20]. Although the computational predictions are still developed, they are often not effective for long RNAs with complex structural motifs and usually do not to take into account physiological conditions and cellular factors that can impact RNA folding [21]. However, the advancement of techniques for enzymatic and chemical probing of RNA structure and incorporation of experimental-based data into the folding algorithms allowed to significantly improve the accuracy of computational RNA structure predictions [22,23,24]. Nowadays, RNA probing techniques rely mostly on chemical modifications of RNA in a base- or sugar-specific way, which are detected by the reverse transcriptase enzyme (RT), which either truncates synthetized cDNA one nucleotide before or inserts the mutation at the site of modifications on the RNA strand [13,21,25]. Coupling of RNA chemical probing with next generation sequencing and advanced bioinformatics tools expands the number of available protocols for the high-throughput effective studying of RNA structure in vitro as well as in vivo (Table 1) [10,26,27,28,29]. For in cell RNA structural studies, the ability of chemicals to penetrate the cell membrane is crucial. To date, there are only few reagents that can be used for RNA secondary structure determination in vivo [30]. Among them, DMS (dimethyl sulfate) and reagents developed for the SHAPE method (selective 2′-hydroxyl acylation analyzed by primer extension) are most widely used in studies in vivo. DMS modifies unpaired adenine and cytosine, but uracil and guanine remain without structural information [3,31,32]. SHAPE reagents act in a base-independent manner and provide structural data for all four nucleotide residues [25,33,34]. Moreover, the methods for incorporation of SHAPE and DMS probing data into RNA structure prediction algorithms are well established [22,35,36].

## 3. The Correlation between mRNA Structure and Translation

**Figure 2:**
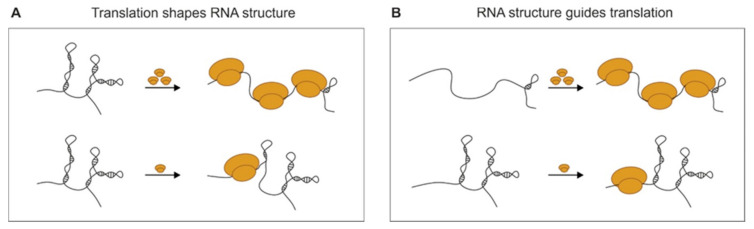
Schematic representation of the interplay between the translation and RNA structure. (**A**) Translation shapes the RNA structure in the cells by ribosome helicase activity and (**B**) the RNA structure guides translation by modulating ribosome binding.

In addition to their protein-coding functions, mRNAs contain cis-acting sequences with a specific secondary structure that may regulate the translation efficiency (TE). The interplay between the translation and RNA structure constitutes the subject of discussion, and it remains an open question whether the RNA structure guides translation or whether translation guides the RNA structure in cells (Table 2 and Figure 2). In general, stable mRNA structural elements tend to reduce the rate of TE, probably by hampering ribosome binding or slowing down its movement [6,40]; however, on the other hand, the ribosome possesses a helicase activity [47], and has been proposed as a major remodeler of the mRNA structure in cells [39,43].

### 3.1. The 5′ and 3′ UTRs

Numerous studies have shown that the stable structures in the 5′ UTR and near the start codon of mRNA can repress the effective initiation of translation in bacteria and eukaryotes [54,55]. The first parallel analysis of RNA structure (PARS; which combines digestion of RNA fragments in vitro by structure-specific enzymes with deep sequencing) on *E. coli* transcriptome complemented with ribosome profiling has indicated that the unstructured sequence upstream of the start codon is a general feature of *E. coli* genes and the secondary structure in this region is negatively correlated with gene expression [45]. Consistent findings were found in recent SHAPE-MaP experiments in living *E. coli* cells that showed that the translation efficiency is regulated by unfolding kinetics of structures overlapping the ribosome binding site (RBS) [43]. According to the RBS kinetic unfolding model, genes with unstructured RBSs have high TE, whereas low TE was observed for those with highly structured RBS. In contrast, DMS-seq analysis of the *E. coli* transcriptome in vivo suggests that TE is only weakly correlated with local RBS structure and is rather regulated by structure of the entire coding sequence (CDS) [40] (see below).

A negative correlation between the high structure of cytoplasmic 5′ UTRs and TE was observed in the mouse and human mRNAs studies using icSHAPE [10,37]. In mouse mRNAs, AUG codons are preceded by a 5-nt sequence with increased accessibility, both in vitro and in vivo, suggesting that the structures around translational start sites are programmed by RNA sequences [10]. Consistent findings have been reported in structural studies of the *Arabidopsis thaliana* transcriptome [8]. By applying structure-seq protocol, they showed that decreases in the average mRNA structure near the start codon facilitate ribosome binding and the start of the translation machinery. The structures that 5′ UTRs fold into may regulate both the cap-dependent and cap-independent initiation of translation [54]. A variety of higher-order RNA structures in the cap region, including pseudoknots, hairpins, and RNA G-quadruplexes, tend to inhibit translation [56,57]. However, cap-independent regulatory RNA structures, including IRES (internal ribosome binding site) or eIF3-binding stem-loop structures, can stimulate translation by promoting loading the translation machinery on the mRNA [58,59,60].

The direct influence of the structural elements within 3′ UTRs on translation remains incompletely discovered, and these structures are usually explored in the context of RNA stability (see below). Nevertheless, the RNA stability and translation efficiency are undeniably inseparable and very often, factors, including 3′ UTR binding proteins that control transcript stability and decay, are also engaged in TE regulation [61,62,63].

### 3.2. The Coding Sequence (CDS)

In general, mRNAs have a lower structure in cells than in vitro, but different RNA structural patterns between species have been found. Across *E. coli* transcripts, especially the coding regions of mRNA tend to be less structured in vivo [40,43]. A strong destabilization of the CDS was also observed in zebrafish in vivo [39]. In addition, global profiling of deproteinized mouse transcripts showed lower degree of structure in CDS compared with UTRs [46]. In contrast, in *Arabidopsis thaliana* and yeast, the average in vivo structure of CDSs was higher or not distinguishable from the UTRs, respectively [8,9]. Additionally, in rice (*Oryza sativa*), the structure of the CDS regions is higher than 3′ UTR, but lower than 5′ UTR [39,42].

The correlation between CDS structure and ribosome density was not found in the PARS study of *E. coli* transcriptome in vitro [45], but the transcriptome-wide analyses in vivo based on DMS-seq or SHAPE-MaP and the ribosome profiling in *E. coli* showed a strong negative correlation between the mRNA structure and TE [40,43]. In cells, the highly translated ORFs appear to have a lower RNA structure and, inversely, those poorly translated ORFs exhibit a high level of RNA structure. In addition, these studies have shown that inhibition of translation (by kasugamycin treatment) leads to stabilization of CDS structure and the greatest structural difference tends to appear precisely in highly translated genes. These observations thus suggest that the ribosome-induced unfolding contributes to mRNA structure destabilization in cells. Mustoe et al. have also found that the correlation between TE and the structure of CDS in vivo is much weaker than those between RBS structure and TE and proposed that TE is rather unaffected by downstream CDS structure (see also Section 3.1). In contrast, the Burkhardt et al. study pointed to the critical role of the intrinsic CDS structure in TE tuning, whereas other features, such as secondary structure and the strength of the RBS, or the codon usage, are only weakly correlated with mRNA translatability [40]. Furthermore, ORF mRNAs have been shown to have modular structures, and the structures and TE of adjacent ORFs inside the one operon can differ significantly.

A strong correlation between the RNA structure and TE was also observed in transcriptome-wide DMS-seq analysis of zebrafish embryos during maternal to zygotic transition (MZT) [39]. A decreased TE of the maternal transcripts has been found to be connected to reduced DMS reactivity, suggesting an increase in the RNA structure and vice versa; transcripts with a higher TE rate were less structured. In addition, in highly translated mRNAs, the CDS regions are much more accessible than 5′ UTRs. Moreover, in embryos treated with an inhibitor of translation initiation (pateamine A), both TE and CDS accessibility to DMS significantly decreased. However, in embryos treated with an inhibitor of translation elongation (cycloheximide), such an effect was not observed. These findings suggest that the initiation of translation and ribosomal entry are crucial for unwinding mRNA into less stable, more open structures. A recent study (icSHAPE) further supports that translation efficiency is correlated with mRNA unfolding in zebrafish embryos [38]. Together, existing data indicate that, at least in some cases, ribosomes have a profound effect on the RNA architecture, and the inherent structure of the coding region appears to have little effect on the translation efficiency.

In contrast to the above studies, the early genome-wide DMS-seq RNA structure analysis in yeast suggested the global unfolding of RNA in vivo, which is not correlated with translation machinery [9]. In this study, the average in vivo RNA structure of CDS does not differ from the structure of UTRs, and a decrease in the RNA structure is not linked to ribosome occupancy, thus suggesting that the translation and RNA structure are not correlated in yeast. Based on the observation of the RNA structure stabilization under ATP-depleted conditions, the authors proposed the strong contribution of energy-dependent factors, such as ATP-dependent helicases in the unfolding of yeast mRNA. Nevertheless, translation is a one of the main energy-consuming processes, and ATP-depletion can also inhibit the action of the ribosomes [64,65].

Furthermore, a recent study of mammalian RNA structures across subcellular compartments has shown that the majority of the transcripts preserve their structure as they transfer from chromatin to the nucleoplasm and cytoplasm [37]. This suggests that the intrinsic RNA structure plays a central role in connecting transcription, translation, and RNA degradation. Although a general trend was observed, where RNA with a lower TE rate tends to be more structured, this study challenges the link between CDS structure and translation efficiency, and highlights the role of RNA binding proteins and RNA posttranscriptional modifications for local RNA structural changes. In contrast, structural differences between nuclear and cytosolic mRNAs were found in the genome-wide analysis of the *A. thaliana* transcriptome [8,41].

An intriguing feature of the coding region detected in diverse organisms in vivo, including, mouse, *Arabidopsis thaliana*, and rice (*Oryza sativa*), is a three-nucleotide periodicity in the mRNA secondary structure [8,10,42,66]. These studies have found that this periodic repeat pattern is significantly associated with ribosome density in vivo, and thus could facilitate translation. However, a three-nucleotide periodicity was not observed in the recent *E. coli* transcriptome-wide study [43].

## 4. RNA Structure in Relation to RNA Stability and Degradation

The stability and fate of mRNA molecules in the cell are under strict control, and at the end of their life cycle, RNAs undergo a carefully regulated degradation. The cap at the 5′-end and the poly(A) tail at the 3′-end are considered as major determinants of mRNA stability, but it can be also regulated by the other intrinsic features of transcripts, such as the sequence, length, and structure of UTRs and RNA modifications in UTRs [3,48,49,67,68]. Transcriptome-wide studies in vivo provide a growing number of evidence that RNA structural features can govern the RNA lifespan (Table 2). The sequences located in UTRs constitute binding sites for diverse trans-acting cellular factors, including RNA-binding proteins (RBPs) and microRNAs [69,70], and the alterations in structure of UTRs might impact their binding and stability of RNA in either positive or negative way [71,72]. The length of mRNA is negatively correlated with its stability in cell and longer transcripts have shorter half-lives in many species [48,73].

In human and yeast, the structure of 5′ UTRs is negatively correlated with mRNA stability, whereas the secondary structure in 3′ UTRs is associated with longer half-lives and higher abundance of mRNAs [44,50,68]. The stable structural elements in the 3′ UTR may block the exosome complex that mainly account for RNA degradation in eukaryotes [74]. In human cells, mRNA 3′-ends tend to be less unfolded than other mRNA regions and specific structure of the 3′-end can facilitate cleavage and polyadenylation of human mRNAs by juxtaposing poly(A) signals (PASs) and cleavage sites that are otherwise too far apart [50]. Interestingly, another study found a negative correlation between RNA structure and RNA half-life in human and mouse cells: more-structured RNAs tended to have shorter half-lives in both the nucleus and cytoplasm [37]. They also proposed that the RNA degradation is not RNA-region specific since the same trends were observed in 5′ UTRs, CDS, and 3′ UTRs.

In contrast to human mRNAs, in yeast, RNA structure content in 3′-ends is similar to other mRNA regions [9]. However, a global analysis of the clusters of yeast mRNA isoforms with different half-lives showed that the double-stranded structures at the 3′-ends, involving or not involving poly(A) tails, are a critical determinant of mRNA stability in yeast [48]. It was also found that the formation of a stable polyU–poly(A) stem-loop can inhibit the association of poly(A)-binding proteins (e.g., Pab1, Ski2, and Xrn1) and lead to increased mRNA stability. Similar findings were obtained in a more recent study of yeast transcriptome, which confirmed the correlation between structure of the 3′-end and mRNA isoform stability, and showed that even closely related mRNA isoforms can form radically different structures in the 3′ UTR in vivo, and they can occur far from the poly(A) site. This study also showed that single-strandedness in the proximity of 3′-end, double-strandedness of the poly(A) tail, together with low Pab1 binding, are linked with mRNA stability and are evolutionarily conserved [49].

An important role of the 3′-end structure for mRNA stability was also observed in zebrafish in vivo. The 3′ UTRs of zebrafish mRNAs are structurally dynamic and changes in their structure can regulate the stability of mRNAs during MZT by modulating miRNA activity [39]. In addition, the 3′ UTRs of zebrafish mRNAs are enriched in cis-acting regulatory elements that control mRNA decay during MZT and have been characterized in detail using a high-throughput RNA-element selection assay (RESA), which enables to identify sequences regulating RNA stability with near nucleotide resolution [75]. An in vivo study of heat-regulated RNA structuromes in rice showed that mRNA unfolding is correlated with RNA half-life and supports the importance of both UTRs for mRNA stability in plants [52]. It was found that heat-induced structural change is greater in UTRs than in other RNA regions and unfolding of 5′- and 3′- ends facilitates access to the RNA degradation machinery.

Moreover, genome-wide RNA decay analysis indicates that codon optimality is also critical for mRNA stability in many organisms, including *E. coli* [76], yeast [77], zebrafish [78], *D. melanogaster* [79], and human [80]. The stable mRNAs are enriched in optimal codons, whereas less stable mRNAs contain predominately non-optimal codons and possess shorter poly(A)-tails.

## 5. The Relationship between RNA Structure and Proteins Binding

Modern technologies combining high-throughput sequencing with in vivo UV crosslinking and RNA immunoprecipitation (e.g., CLIP-seq, RIP-seq, and RIP-Chip), together with new computational approaches provided new insights into the landscape of the RNA binding proteome [81]. To date, 1753 proteins have been identified in the human RNA-binding proteome, including 978 proteins interacting with poly(A) RNA and 775 proteins that bind non-poly(A) RNA, highlighting the complexity of RNA–protein interactions in vivo [82]. RNA-binding proteins (RBPs) coordinate all of the essential cellular processes, and are an indispensable element in the co- and post-transcriptional regulation of mRNAs and ncRNAs. Various RBPs can dynamically bind RNAs across cellular compartments and during different steps of the lifecycle, including RNA transcription, post-transcriptional processing, translation, stability, and decay [70,83]. A comparative analysis of the human and yeast RNA-binding proteome showed that the RNA-binding activity in vivo and the structural features of many RBPs are strongly conserved in Eukaryotes [84].

Many RBPs need to recognize a specific RNA sequence or structure for their function in a cell [85,86,87], but other proteins can bind RNA in nonspecific manner, including diverse cellular and viral RNA chaperones that can remodel the RNA structure and facilitate interactions with other partners, as has been shown in studies in vitro [88,89]. However, the regulatory networks between the cellular transcriptome and proteome are only beginning to be understood, and little is known about the target RNA sequences and structural preferences of RBPs in vivo. Genome-wide studies of the sophisticated interplay in the RNA interactome in vivo are still challenging, as RNA–protein complexes tend to be dynamic and change during the RNA lifetime. Advanced global studies correlate the large-scale studies of the RNA-binding proteome with structural information from the transcriptome-wide RNA structure probing in vivo. They confirm the binding of RBPs to specific sequences in the 5′ and 3′ UTRs, and the critical role of these interactions for the initiation of translation, RNA processing, and its stability in cells (Table 2, and see above). These studies also show that a significant fraction of RNA-binding motifs are present in the coding region and introns [90,91]. The association of individual RNA structures within *S. cerevisiae* transcripts with their interacting proteins revealed that many RBPs recognize evolutionary conserved RNA structures in CDS, possibly formed by the degenerated codons [90]. These interactions have been proposed to regulate post-transcriptional processes, such as tRNA binding and ribosomal biosynthesis, or that these RBPs may act as metabolic enzymes or kinases.

A recent investigation of human transcriptome focused on the interplay between the structure of RNA and its ability to facilitate protein binding revealed a relationship called the RNA structure-driven protein interactivity, which has an important functional role [53]. According to this theory, the structural content in RNA molecules regulates the number of protein bindings. RBPs interact more with the highly structured RNAs that are rich in double-stranded regions, whereas an opposite trend has been found for poorly structured transcripts. Furthermore, highly structured transcripts preferentially bind polypeptides and encode the regulatory proteins involved in a large number of cellular networks. These findings indicate functional differences between highly and poorly structured RNAs, and suggest the existence of a new, sophisticated layer of post-transcriptional regulation of genes expression. Although this relationship needs to be more closely investigated, a recent comparative analysis of 114 in vivo RBP interaction maps from multiple PAR-CLIP experiments performed in HEK293 cells identified the modules of RBPs that are constituted by subsets of proteins that preferentially bind to specific sets of RNAs and targeted regions, and possibly play role in posttranscriptional regulation [91].

In contrast, other genome-wide studies in mammalian cells point to the contribution of RBPs to the RNA structural rearrangements that are distinct from ribosome—or ATP-helicase induced RNA unwinding [10,37]. As diverse subsets of RBPs can bind RNA in specific cellular compartments, they have been proposed to account for local RNA structural changes observed between chromatin, nucleoplasm, and cytoplasm [37]. For example, the heterogeneous nuclear ribonucleoprotein C (HNRNPC) splicing factor, which preferentially recognizes single-stranded uridine tracts [92], is directly involved in the destabilization of the RNA structure around its binding site that, together with m^6^A modification, facilitates HNRNPC-binding in the chromatin RNA fraction [37]. In contrast, the Staufen homolog I, which is a double-stranded-binding RBP involved in the transport and localization of mRNAs to different subcellular compartments [93], seems to participate in stabilizing the RNA structure after RNA release from the chromatin [37].

## 6. Impact of RNA Modifications on RNA Structure In Vivo

As the RNA, in its life cycle, undergoes numerous post-transcriptional modifications, adding various chemical groups to their bases can significantly influence the RNA folding, stability, and interactions with cellular factors [94,95,96]. The aberration of RNA modification patterns has been associated with various diseases in human, such as cancerogenesis [97]. Among the hundreds of possible RNA chemical modifications, the most abundant across the mRNA is N^6^-methyladenosine (m^6^A). Thus, it is not surprising that m^6^A modification is of particular interest in research. Development of an m^6^A RNA immunoprecipitation approach followed by high-throughput sequencing (MeRIP–seq) allowed to study m^6^A modification landscape in a transcriptome-wide manner [98,99]. These studies identify over 12,000 m^6^A sites, mainly in the context of the sequence GGm^6^ACU, in more than 7000 human transcripts. The sites of m^6^A modification are highly conserved between humans and mice and preferentially appear in internal long exons, around the stop codons, and in the 3′ UTRs. However, the exact functions and, in particular, the effects on RNA folding are still not completely understood. The regulatory function of m^6^A mRNA modification has been shown in transcription, splicing, mRNA export and stability, and translation [37,99,100,101,102]. It can also impact various physiological processes, such as the clearance of maternal mRNAs during zebrafish MZT [103], mammalian cortical neurogenesis [104], and plays regulatory role in human cancer [105,106].

Thermodynamic study has shown that m^6^A influences the RNA structure because of the rotation of the methlyloamino group, from syn to anti conformation, with a higher energy, thus destabilizing the RNA duplexes by 0.5–1.7 kcal/mol [51]. The opposite effect was observed in the single stranded region of RNA, where m^6^A can contribute to increasing the stability of the RNA molecule, probably by base stacking. Ex vivo studies of human transcriptome confirmed the structural RNA changes at the m^6^A modification sites, with a strong tendency for unwinding RNA secondary structure [51]. Therefore, the appearance of m^6^A in RNA has been proposed to work as a “molecular switch” for the RNA structure [107]. Spitale et al. first comprised the in vivo SHAPE reactivities of m^6^A-modified vs. unmodified transcripts, and showed that in the cell RNA regions, both surrounding and including the modified A residues, tend to be unpaired [10]. To check whether decreased base-pairing at the modification sites is caused by the m^6^A destabilizing effect on RNA duplexes or the modification machinery preferentially methylate adenosine at single-stranded sites of RNA, they performed genetic knockout of N^6^-adenosine-methyltransferase (*Mettl3*) in mouse ES cells. The depletion of *Mettl3* led to a transcriptome-wide reduction of the SHAPE signal at the m^6^A modification sites, confirming the RNA destabilizing properties of m^6^A modification in vivo.

Recent transcriptome-wide studies across the three different cellular compartments in mammalian cells further supported that m^6^A enriched regions in transcripts are far less structured than the same, but unmethylated, sites of RNAs, and the patterns of m^6^A-induced structural destabilization are similar in chromatin, nucleoplasm, and cytoplasm [37]. However, the RNA structure is more open after RNA release from the chromatin to nucleoplasm, consistent with *METTL3-METTL14* complex’s localization to specific nuclear loci [102,108]. Furthermore, across all of the analyzed compartments, RNA modifications significantly overlap with both structural-change sites and RBP bindings, suggesting that many RBPs require induced by the m^6^A local destabilization of RNA for their binding. For example, in the chromatin fraction, m^6^A modification can facilitate the binding of HNRNPC by the disruption of the local RNA secondary structure in close proximity to the binding sites [37,107]. In addition, m^6^A can work as an “RNA molecular switch” in plants [42]. However, the significant correlation between the structural changes and m^6^A in rice has been observed only in 3′ UTR and not in the CDS or 5′ UTR regions.

## 7. Conclusions and Future Perspectives

Recent transcriptome-wide studies have significantly increased the knowledge about the interplay between the RNA secondary structure and RNA functions in cells. They allow for considering the influence of various cellular factors on RNA folding in vivo, and vice versa, as well as the impact of the RNA structure on critical biological processes. However, there are still many unanswered questions and challenges. For example, it remains unclear whether the RNA folding pattern is species-specific or observed differences result from application of various RNA structure probing methods. An important area for advancement is further development of methods for more accurate studying of the coexisting RNA conformers, RNA co-transcriptional folding, and differentiation of intramolecular RNA interactions and intermolecular RNA–protein or RNA–RNA bindings. Together, with continuous experimental/technical development, there is also a need to advance the computational tools not only for high-throughput data analysis, but also for the experimentally supported accurate modeling of the RNA structure in the native in vivo form.

## Figures and Tables

**Table 1:** High-Throughput RNA Structure Probing Methods Used in Studies Described in this Review.

Method	Application Described in this Review	Used Probe	Modification Readout	Condition
icSHAPE	Mouse [10,37], human [37], zebrafish [38]	NAI-N_3_	RT-stop	In vivo and in vitro
DMS-seq	Yeast [9], zebrafish [39], *E. coli* [40]	DMS	RT-stop	In vivo and in vitro
SHAPE-Structure-seq	*A. thaliana* [41]	NAI	RT-stop	In vivo and in vitro
Structure-seq	*A. thaliana* [8], rice [42]	DMS	RT-stop	In vivo
SHAPE-MaP	*E. coli* [43]	1M7	RT-mutate	In vivo and in vitro
SPLASH	Human and yeast [44]	Biotinylated psoralen	Mapping of ligated junctions	In vivo
PARS	*E. coli* [45]	RNase V1 (dsRNA) and S1 (ssRNA)	Fragments analysis	In vitro
CIRS-seq	Mouse [46]	DMS and CMCT	RT-stop	In vitro

1M7, 1-methyl-7-nitroisatoic anhydride; CIRS, chemical inference of RNA structures; CMCT, N-cyclohexyl-N-(2-morpholinoethyl) carbodiimide metho-p-toluene sulfonate; DMS, dimethyl sulfate; icSHAPE, in vivo click SHAPE; NAI, 2-methylnicotinic acid imidazolide; NAI-N3, 2-(azidomethyl)nicotinic acid acyl imidazole; PARS, parallel analysis of RNA structure, RT, reverse transcriptase; SHAPE-MaP, selective 2′-hydroxyl acylation analyzed by primer extension and mutational profiling; SPLASH, sequencing of psoralen crosslinked, ligated, and selected hybrids.

**Table 2:** The Critical Conclusions from Reviewed Transcriptome-Wide Studies.

Work	Organism	Main Conclusions
Del Campo et al., 2015 [45]	*E. coli*	The unstructured sequence upstream of the start codon is a general feature of *E. coli* genes and is positively correlated with gene expression.
Mustoe et al., 2018 [43]	*E. coli*	Translation is the main source of mRNA structural destabilization in cells. The structure in RBS is a strong determinant of TE. CDS structure is not critical for TE.
Burkhardt et al., 2017 [40]	*E. coli*	The structure in RBS does not determine TE. The intrinsic CDS structure plays the critical role in TE tuning.
Beaudoin et al., 2018 [39]	Zebrafish	Translation guides RNA structure rather than structure guiding translation. The ribosome is a major remodeler of RNA structure. Structural elements in the 3′ UTR are major regulators of transcript stability during the MZT.
Shi et al., 2020 [38]	Zebrafish	TE is correlated with RNA unfolding.
Rouskin et al., 2014 [9]	Yeast	ATP-dependent processes strongly contribute to the unfolded state of mRNAs inside cells.
Geisberg et al., 2014 [48]	Yeast	The double-stranded structures at the 3′-ends, involving or not involving poly(A) tails, are a critical determinant of mRNA stability.
Moqtaderi et al., 2018 [49]	Yeast	The single-strandedness in the proximity of 3′-end, double-strandedness of the poly(A) tail, together with low Pab1 binding, are linked with mRNA stability and are evolutionarily conserved.
Aw et al., 2016 [44]	Human and yeast	The structure of 5′-UTRs is negatively correlated with mRNA stability, whereas the secondary structure in 3′ UTRs is associated with longer mRNA half-life.
Wu et al., 2017 [50]	Human	In cells, 3′-ends are generally more folded than are other mRNA regions and their structure regulates mRNA metabolic stability. Specific structure of the 3′-end can facilitate cleavage and polyadenylation of mRNAs.
Roost et al., 2015 [51]	Human	Ex vivo studies of human transcriptome confirmed the structural RNA changes at the m^6^A modification sites, with a strong tendency for unwinding RNA secondary structure.
Sun et al., 2019 [37]	Mouse and human	The intrinsic RNA structure plays a central role in connecting transcription, translation, and RNA degradation. The majority of the transcripts preserve their structure as they transfer from chromatin to the nucleoplasm and cytoplasm. RBPs and RNA modifications account for local RNA structure changes between cellular compartments. CDS structure and TE are only weakly correlated. More-structured RNAs tended to have shorter half-lives. RNA degradation is not RNA-region specific.
Spitale et al., 2015 [10]	Mouse	m^6^A modifications impact RNA structure in vivo, favoring the transition from paired to unpaired RNA.
Deng et al., 2018 [42]	Rice	Higher m^6^A modification tends to have less RNA structure in the 3′ UTR in plants.
Su et al., 2018 [52]	Rice	Transcripts are subjected to degradation by a mechanism involving secondary structure unfolding in 5′ and 3′ UTRs.
Ding et al., 2014 [8]	*A. thaliana*	Less structured regions immediately upstream the start codon region facilitate ribosome binding and increase TE.
Liu et al., 2019 [41]	*A. thaliana*	Nuclear mRNAs fold differently from cytosolic mRNAs.
Sanchez De Groot et al., 2019 [53]	Various	Highly structured RNAs bind a large amount of proteins.
